# State-Dependent Brain Stimulation for Visual Neurorehabilitation: Principles and Applications

**DOI:** 10.3390/vision9030050

**Published:** 2025-06-20

**Authors:** Kuzma Strelnikov, Juha Silvanto

**Affiliations:** 1 Centre for Cognitive and Brain Sciences, University of Macau, Macau SAR 999078, China; 2 Department of Public Health and Medicinal Administration, Faculty of Health Sciences, University of Macau, Macau SAR 999078, China; 3 Department of Psychology, Faculty of Social Sciences, University of Macau, Macau SAR 999078, China

**Keywords:** noninvasive brain stimulation, transcranial magnetic stimulation (TMS), state-dependent, rehabilitation, cortical blindness, amblyopia

## Abstract

The effects of Transcranial Magnetic Stimulation (TMS) depend on stimulation parameters such as intensity, location, frequency, and duration. In clinical practice, these parameters are often adapted from studies carried out in healthy individuals. However, in this narrative review, we indicate that the impact of TMS is also highly state-dependent, meaning it is influenced by the excitability of the targeted brain region at the time of stimulation. This state-dependency complicates the translation of findings from healthy individuals to clinical populations, as neurological disorders often alter brain states, limiting the applicability of standard stimulation protocols. To address this challenge, stimulation parameters must be chosen within a framework that accounts for the interaction between external stimulation and the brain’s internal state. Such an approach enhances the specificity of interventions, allowing for targeted modulation of neural populations by manipulating brain states prior to stimulation. State-dependent TMS has shown promise in conditions like cortical blindness and amblyopia, where tailored approaches based on the brain state associated with the condition have facilitated more precise and effective treatments. We advocate that integrating state-dependent knowledge tailored to the specifics of visual disorders alongside judicious selection of stimulation parameters holds the potential to establish a comprehensive paradigm for future investigations.

## 1. Introduction

Methodological discussions around non-invasive brain stimulation often focus on the physical stimulation parameters, such as intensity, location, frequency, and duration of stimulation. In translational research, these parameters are typically chosen based on their effectiveness in inducing behavioral or neural changes in healthy participants. For instance, repetitive transcranial magnetic stimulation (rTMS) protocols commonly use low (e.g., 1 Hz) or high (e.g., 10 Hz) frequencies, believed to induce long-term depression (LTD) or long-term potentiation (LTP), respectively (e.g., [1,2]). The standard approach involves identifying the optimal parameters and applying them to specific clinical conditions.

However, there are two major challenges with this approach. First, there is no universally ideal combination of stimulation parameters, as their effects depend on nonlinear interactions, not only between the parameters themselves but also with the brain’s state. Neural excitability, influenced by numerous factors, can cause the same brain stimulation protocol to produce facilitatory effects in one context but inhibitory effects in another (see, e.g., [3] for review). Second, translating basic research to clinical applications is complicated by the altered brain states associated with neurological conditions.

This review explores the application of state-dependent brain stimulation in treating clinical conditions affecting the visual system, with a primary focus on TMS due to its well-documented mechanisms of state-dependency. We focused on visual disorders with previous research on state-dependent methods. Other than these pre-requisites, we selected relatively common and well-known illnesses. The discussion begins with a review of key findings and principles of state-dependency, followed by an examination of its clinical applications, emphasizing two conditions where tailoring stimulation parameters to underlying brain state has shown significant impact: cortical blindness and amblyopia.

## 2. State-Dependent Brain Stimulation: Noninvasive “Microstimulation”

In basic cognitive neuroscience research, TMS has traditionally been regarded as a tool for “disrupting” brain activity (see [4] for a recent review). This approach, often referred to as “virtual neuropsychology,” involves using TMS to temporarily disrupt brain activity in order to assess its causal role in perceptual and cognitive functions. Early TMS studies frequently focused on the visual domain; indeed, the first demonstration of TMS applications beyond the motor cortex targeted the visual cortex [5]. In this seminal study, single TMS pulses were applied to the calcarine cortex at intervals from 0 to 200 ms with respect to stimulus (letters) presentation, which participants were asked to report. When the onset asynchrony between the visual stimulus and the TMS pulse was 80–100 msec, detection performance was reduced. Subsequent studies in the 1990s extended these findings, demonstrating selective effects on motion perception when the motion-sensitive area V5/MT was stimulated (e.g., [6]). Today, there is a substantial body of literature on the use of “virtual lesion” TMS to study the visual cortex (see [4]).

**Figure 1:**
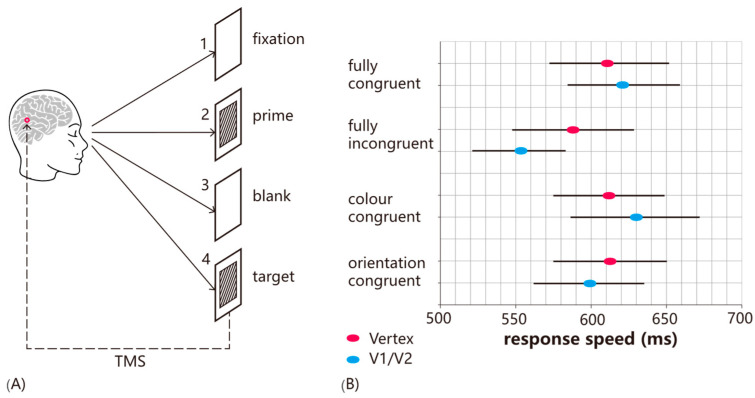
State-dependent effects of TMS in a visual priming task. (**A**) Experimental design. Participants were shown a prime stimulus—a red–black or green–black grating, tilted either clockwise or counterclockwise—followed by a target stimulus. The target could be fully congruent with the prime (matching in both color and orientation), fully incongruent (differing in both attributes), or partially congruent (matching either in color or orientation). Participants were tasked with identifying the color of the target’s diagonal lines (red or green). Single-pulse TMS was applied 100 milliseconds after the target’s onset, either over V1/V2 or the vertex (serving as a baseline control, adapted from [10]). (**B**) Results. Compared to vertex stimulation, TMS applied over V1/V2 caused a significant delay in response time for correctly identifying fully incongruent targets. However, TMS had no effect on response times for fully or partially congruent targets. This highlights the specificity of TMS effects, influenced by the congruence between the prime and target stimuli.

The state-dependent approach is an attempt to move beyond virtual neuropsychology by conceptualizing the effects of brain stimulation as an interaction between ongoing brain activity and the stimulation parameters. In the visual domain, this principle was first systematically explored by combining TMS with psychophysical manipulations such as visual adaptation or priming. Preconditioning through these methods produces highly specific behavioral outcomes, determined by the nature of the preconditioning itself [7,8]. In a study by [9], participants were adapted to a uniform red color for 30 s before phosphenes were induced by stimulating the early visual cortex. The induced phosphenes matched the color of the adapting stimulus, indicating that TMS had selectively facilitated the detection of the adapted attribute. In a related experiment, participants were primed by a combination of orientation and color (see Figure 1). TMS was found to impair the detection of primed targets, whereas other target types were unaffected, demonstrating the increasing specificity provided by the state-dependent approach (as without the priming manipulation, TMS would impair all targets equally).

**Figure 2:**
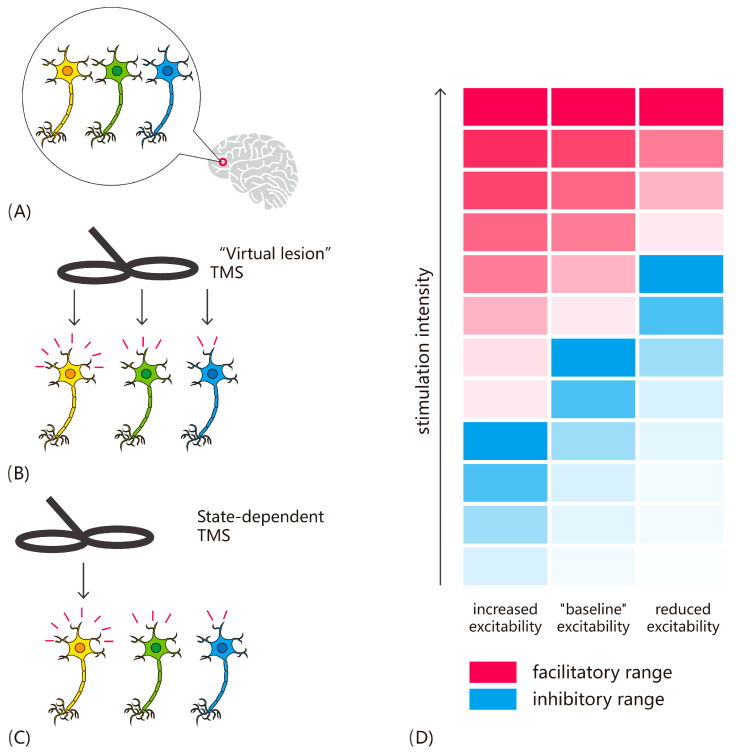
Principles of state-dependent TMS as “noninvasive microstimulation”. (**A**) Cortical regions comprise neurons with diverse tunings and selectivities, presenting a challenge in visual neuroscience: understanding the unique responses of these populations and how they combine to shape perception. (**B**) Traditional “virtual lesion” TMS assumes that all neurons in the targeted area are indiscriminately stimulated. While this approach can reveal whether a brain area is involved in visual perception, it cannot provide insight into the contributions of differently tuned neuronal populations. (**C**) State-dependent TMS overcomes this limitation by leveraging differences in neuronal excitability. Neural populations are brought into distinct excitability states, modifying their susceptibility to stimulation. This enables targeted stimulation of functionally distinct neuronal groups within the same area. (**D**) Noninvasive brain stimulation techniques such as TMS induce specific behavioral and neural facilitation ranges based on the applied current intensity. These ranges are further influenced by the excitability state of the neurons. Consequently, the same TMS intensity can produce either facilitation or impairment, depending on the neurons’ baseline excitability. Adapted from [13].

Thus, a key feature of state-dependent manipulations is that they allow us to more selectively estimate specific subpopulations of neurons (see Figure 2). Cortical regions can contain a wide range of neurons with distinct tunings, and in the “conventional” approach, it is assumed that these are all stimulated randomly. However, with state manipulations such as adaptation, the differential susceptibility to TMS as a function of brain state can be leveraged, effectively allowing TMS to be used akin to “microstimulation”. For example, whereas in a typical “virtual lesion” study, TMS applied to the motion-sensitive area V5/MT would affect motion perception generally, with adaptation/priming approaches, it is possible to target specific directions of motion (e.g., [11,12]).

## 3. Stimulation Intensity and Brain State

Stimulation intensity has been consistently found to modulate the direction of TMS effect both behaviorally and at the neural level. For instance, Ref. [14] demonstrated that low-intensity TMS enhances neural activity and visually induced neural firing for up to 200 ms, while higher TMS intensities reverse this facilitation, resulting in suppressed neural activity. In contrast, studies on TMS-induced visual masking have shown that the intensity required to impair the detection of external stimuli is higher than the intensity needed to produce phosphenes. Typically, impairments in visual detection occur when suprathreshold TMS is applied to the early visual cortex 80–120 ms after a target stimulus appears [15,16,17]. This appears to match well with the suppression observed at the neural level when TMS is applied at a high intensity (cf. [14]).

Furthermore, while suprathreshold TMS is necessary to disrupt the detection of visual stimuli, subthreshold TMS (below the phosphene threshold) can produce qualitatively different effects. Specifically, subthreshold TMS has been shown to facilitate the detection of near-threshold stimuli, enhancing perception within the same time window typically associated with TMS-induced masking [18,19,20]. These findings highlight the importance of TMS intensity in determining its effects on visual processing. However, such effects are observed when the baseline level of performance is low. In a study by [20], participants engaged in a motion direction discrimination task, calibrated to achieve either a low (60%) or high (85%) baseline performance level. During the task, a TMS pulse train (three pulses at 10 Hz) was delivered over the V5/MT region at varying intensities, synchronized with stimulus onset. The results demonstrated that high-intensity TMS impaired performance when participants were operating at a high baseline level, while subthreshold TMS enhanced motion discrimination at the lower baseline level. These findings align with a series of studies by [18,19], which similarly reported performance facilitation with subthreshold TMS. These results are generally explained in terms of stochastic resonance, a known phenomenon whereby adding low-intensity noise to weak signals helps them exceed the perceptual threshold [20].

When considering the effects of TMS on the brain’s neural networks, it is crucial to acknowledge the inherent lack of spatial specificity associated with the technique. Unlike invasive electrophysiological interventions, which can selectively manipulate individual neurons or synapses, TMS typically affects a broader region of cortex, influencing a diverse population of excitatory (glutamatergic), inhibitory (GABAergic), and modulatory neurons (see, e.g., [21,22]). The net effect of TMS on behavior, whether facilitatory or disruptive, emerges from the complex interplay of these neuronal populations, shaped by their existing connections, intrinsic excitability, and ongoing activity patterns (see, e.g., [3,23]). As such, interpreting TMS effects requires considering the dynamic and interconnected nature of neural circuits, rather than simply attributing effects to the direct activation or suppression of a single neuronal type.

The terms “inhibition,” “excitation,” and “facilitation” are often used in literature to describe the behavioral and network-level effects of TMS (see, e.g., [3] for detailed discussion). It is important to distinguish these effects from the underlying mechanisms at the cellular and synaptic level. For instance, “inhibition” at the behavioral level (e.g., impaired performance on a visual task) may arise from a complex combination of factors, including reduced neuronal firing rates in specific cortical regions, increased activity in inhibitory interneurons, or disruption of long-range connections between brain areas (see, e.g., [24]). Similarly, “facilitation” may reflect increased neuronal excitability, enhanced synaptic transmission, or improved coordination between different neural populations. The specific mechanisms underlying these behavioral and network-level effects require deeper investigation using techniques that provide more direct access to neuronal activity along with computational modeling (see, e.g., [25]).

Furthermore, it is important to consider that the effects of stimulation intensity are not independent of brain state. The interaction between TMS intensity, brain state, and timing of stimulation was tested directly using a priming paradigm where participants were primed with a combination of color and orientation and then asked to detect the color of a target grating [10]. Typically, TMS facilitates the detection of non-primed attributes while leaving primed items unaffected. In Experiment 1, single-pulse TMS was applied at target onset over the early visual cortex or vertex at either subthreshold (80% PT) or suprathreshold (120% PT) intensity. At this point, visual input related to the target stimulus has not reached V1/V2, and, thus, TMS effects would reflect an interaction with prime-related activation. Both intensities facilitated incongruent targets, consistent with prior findings. In Experiment 2, TMS was applied 100 ms after target onset, during the classic TMS-masking window when V1/V2 is processing visual input. Here, only suprathreshold TMS facilitated non-primed target detection, with no effect at subthreshold intensity. The likely explanation is that at this time point, V1/V2 neurons were firing in response to visual stimulation, and the subthreshold stimulation was not sufficient to drive the neurons further; in other words, their excitability had decreased. The implication is that, when designing TMS protocols, it is not sufficient to think in terms of stimulation intensities without considering the interaction with ongoing brain activity. Fundamentally, TMS effects depend on a complex interplay of intensity, timing of stimulation, and brain state. To explain these effects mechanistically, ref. [13] proposed a model in which TMS has specific intensity ranges that either enhance or inhibit neural activity and behavior, and these ranges shift depending on changes in neuronal excitability (see Figure 2D). For example, when excitability is reduced, such as through adaptation, the facilitatory and inhibitory ranges shift toward higher stimulation intensities. Conversely, when excitability is increased, such as by attention, lower stimulation intensities can produce similar effects. The implication is that a TMS intensity that facilitates behavior at baseline may become disruptive when excitability increases, due to the shifting facilitatory-inhibitory range.

Furthermore, it is essential to recognize that pathological brains often exhibit altered baseline cortical excitability compared to healthy individuals (e.g., [23,26]). In conditions characterized by reduced excitability, such as areas surrounding a stroke lesion or in some forms of cortical blindness, the facilitatory range of low-intensity TMS may be shifted towards higher intensities. Clinicians should also consider the importance of timing, ensuring stimulation occurs within an appropriate time window to capture the intended neural processes [15,16,17]. The optimal stimulation parameters are inevitably condition-specific.

## 4. State-Dependency in Plasticity-Inducing TMS Paradigms

The studies reviewed above, which demonstrate the basic principles of state-dependency, deal with single pulses or brief pulse trains. In the clinical domain, the most common usage of TMS is in repetitive mode. The most common rTMS paradigms are 1 Hz rTMS and 10 Hz rTMS, which are believed to decrease and increase cortical excitability, respectively [1,2]. However, there is evidence to indicate that these protocols in themselves are neither inhibitory nor facilitatory but can vary depending on the brain state. Indeed, numerous studies have found inhibitory effects of high-frequency rTMS protocols (≥10 Hz), at least when given during a task (e.g., [27] for review in the language system). In the visual system, 1 Hz rTMS (which is generally inhibitory) can facilitate neural processing if the targeted area is in a suppressed state [28].

Furthermore, numerous studies have successfully combined rTMS with concurrent behavioral tasks in a state-dependent manner to enhance the specificity of the aftereffects. One of the earliest examples involved a modified theta burst TMS paradigm, which was paired with visual stimulus presentation, in which participants viewed motion stimuli in a specific direction while receiving TMS [29]. The aftereffects of TMS on subsequent motion-direction discrimination were found to depend on the direction of motion viewed during stimulation: detection of the congruent direction (i.e., direction viewed during offline TMS) was unaffected, whereas detection of the incongruent direction (i.e., opposite direction to the one viewed during offline TMS) was impaired. Thus, TMS appeared to most effectively activate neurons that were at rest (and thus in a higher state of excitability) than neurons tuned to the direction of motion presented during TMS.

Moreover, state-dependent rTMS has been applied in depression treatment through a protocol that involved administering a cognitive task engaging the prefrontal cortex prior to TMS. Ref. [30] built on findings that higher activity in the rostral anterior cingulate cortex (rACC), associated with increased frontal theta power, correlates with better antidepressant outcomes from rTMS. To optimize the therapy, a cognitive task was used to activate frontal theta before delivering the stimulation. Patients who followed this state-dependent protocol experienced significantly greater reductions in depression scores compared to those who received sham TMS with the task or active TMS without it. These results suggest that using cognitive engagement to modulate brain state prior to rTMS may offer a promising strategy for enhancing treatment efficacy in depression (double-blind, randomized, sham-controlled trial, *n* = 12 for each group, effect assessed in two weeks). However, further investigation in larger clinical trials is needed.

In practical clinical settings, assessing cortical excitability or baseline activity can be approached through several methods. Clinical observation and a thorough neurological examination offer a basic level of assessment, revealing signs of hypo- or hyperexcitability (spontaneous movements, heightened reflexes, abnormal sensory thresholds) (see, e.g., [23]). In resting-state EEG, one can observe alterations in dominant frequencies (e.g., increased delta or theta activity suggesting reduced cortical arousal) or abnormal patterns of synchrony. In EEG-evoked potentials, the amplitude and latency of EPs can provide information about excitability (see, e.g., [31]). Using TMS, the motor threshold can be quickly and easily determined, reflecting motor cortex excitability (e.g., [32]). In addition, cognitive and behavioral assessments can offer indirect measures of excitability by assessing reaction time, accuracy, or perceptual thresholds. The most comprehensive assessment would involve combining information from multiple sources and within the patient’s clinical context.

Recent developments allowing TMS to temporarily strengthen connections between cortical areas might be particularly fruitful for the clinical domain. This technique, known as corticocortical paired associative stimulation (ccPAS) [33,34,35], is based on the Hebbian principle according to which synaptic connections are reinforced when presynaptic neurons consistently fire just before postsynaptic neurons [36]. By delivering repeated stimulation to interconnected cortical regions at precisely timed intervals, ccPAS replicates the natural communication patterns between these areas, akin to spike timing-dependent plasticity (STDP) [37].

Conventional ccPAS, like other TMS protocols, is limited by its lack of spatial and temporal specificity, meaning it cannot precisely target connections linked to specific functions. To address this limitation, Ref. [38] developed a new “function-tuning ccPAS” protocol based on the principles of state-dependency. Specifically, during the application of ccPAS over the motion area V5/MT and the early visual cortex, participants viewed a motion stimulus moving in a specific direction. The protocol was found to enhance the detection of the motion direction primed during the visual-TMS pairing. This innovative “function-tuning ccPAS” paradigm thus allowed for selective enhancement of synaptic efficiency in functionally distinct but spatially overlapping pathways between V5/MT and V1/V2. Notably, these effects were observed only at subthreshold, not suprathreshold, intensities, highlighting the critical importance of considering stimulation intensity in conjunction with state-dependent mechanisms. This protocol significantly increased the specificity of TMS-induced plasticity by enabling targeted modulation of cortico-cortical pathways tied to specific functions.

While this review primarily focuses on TMS due to its well-documented mechanisms of state-dependency as well as wider clinical use, one should mention other non-invasive brain stimulation techniques. Transcranial Direct Current Stimulation (tDCS) and Transcranial Alternating Current Stimulation (tACS) are potential alternative approaches with differing strengths and weaknesses (see, e.g., [39] for review). TMS uses magnetic pulses to induce brief electrical currents, enabling precise targeting of specific cortical areas and the modulation of neural activity. tDCS, on the other hand, applies a weak direct current to the scalp, modulating neuronal excitability by shifting the resting membrane potential. tACS delivers an alternating current, aiming to modulate endogenous brain oscillations.

## 5. Implications of State-Dependency for Rehabilitation

A consequence of state-dependency is that any TMS effect represents a snapshot of a specific combination of external and internal factors, and changes in either are likely to alter the resulting aftereffect. Therefore, effective use of TMS requires a theoretical model that captures this interaction; in clinical settings, this necessitates a deep understanding of the neural mechanisms underlying the targeted condition. In the visual domain, brain stimulation techniques are being explored to treat conditions such as amblyopia, post-stroke hemianopia, and central vision loss caused by age-related macular degeneration (see, e.g., [40] for review). While the field is still in its early stages—highlighted by a recent review noting that “variables such as stimulation duration, intensity, and location need further investigation, and no consensus exists on the best rehabilitative approaches for specific visual impairments” [40]—there is growing evidence that state-dependent approaches can enable the development of more effective treatment protocols. Two such examples will be discussed below.

### 5.1. Amblyopia

Amblyopia (“lazy eye”) is the leading cause of monocular visual impairment in adults, affecting around 3% of the population. It is a neurodevelopmental disorder where one eye fails to achieve normal visual acuity due to disrupted visual input during early childhood, despite the absence of structural abnormalities. Standard treatment involves patching or penalizing the stronger eye during childhood; for individuals over the age of 12 who miss this critical period, there are no widely accepted treatment options, highlighting a significant unmet need for effective therapies (e.g., [41]).

Thompson et al. [42] provided the first evidence on the feasibility of using rTMS (applied to the primary visual cortex) to temporarily enhance contrast sensitivity in adults with amblyopia. The study involved nine amblyopic subjects and five control participants with normal vision to investigate the effects of rTMS on amblyopia. In the 1 Hz stimulation condition, seven out of nine patients showed a reliable reduction in contrast sensitivity in the nonamblyopic eye at T2 (30 min after rTMS), with effects not observed reliably in other conditions. Among the six amblyopic participants also tested with 10 Hz stimulation, all showed improved contrast sensitivity at both T1 (just after rTMS) and T2, including those who did not respond to 1 Hz stimulation, indicating a positive effect under these conditions. The ability of TMS to induce a facilitation indicates that amblyopia’s visual deficits are not due to the absence of functional neural circuits but rather their abnormal functioning. The authors also report that the larger the absolute difference between the two eyes at the baseline, the greater the effect of rTMS on the amblyopic eye. However, the duration of the effect was not estimated. The effectiveness of both 1 Hz (typically considered inhibitory) and 10 Hz (typically excitatory) protocols underscores the prior point that the effects of rTMS are not strictly determined by stimulation frequency, challenging the conventional categorization of low- vs. high-frequency protocols as inherently inhibitory or excitatory. It is possible that low- and high-frequency TMS induced a benefit via distinct mechanisms, by facilitating the representations associated with the amblyopic and healthy eye and suppressing those associated with the healthy eye. The outcome in both cases is rebalancing the excitability of neural populations in the visual cortex. Molecular and histological investigations in amblyopic rats revealed that rTMS, compared to sham stimulation, significantly increased expression of synaptic plasticity genes and dendritic spine density, while simultaneously reducing inhibition and the density of perineuronal nets in the contralateral visual cortex to the deprived eye visual cortex [43].

**Figure 3:**
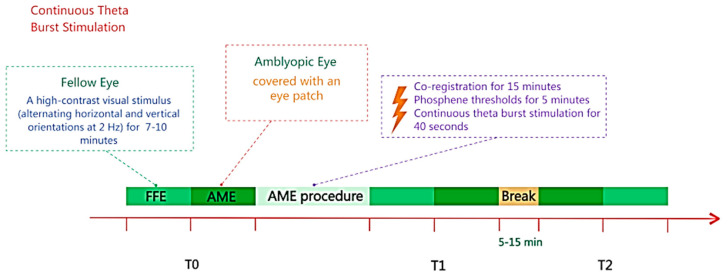
State-dependent TMS for improving amblyopia [44]. During the application of cTBS, participants viewed a high-contrast visual stimulus (alternating horizontal and vertical orientations at 2 Hz) with their fellow eye (FFE), while the amblyopic eye (AME) was covered with an eye patch. Sustained improvements in contrast sensitivity were observed following five consecutive daily cTBS sessions (re-assessments conducted at 8, 19, and 78 days post-stimulation.

Moreover, Clavagnier et al. [44] developed a state-dependent approach based on the theory that deficits in amblyopia are associated with suppressed neural circuits (see Figure 3). The objective was to direct the effects of continuous theta burst stimulation (cTBS) toward suppressed neurons associated with the amblyopic eye. This was achieved by having five participants view high-contrast stimuli with their non-amblyopic eye during stimulation, leveraging the principle that suppressed neuronal populations are preferentially activated by TMS. While cTBS is generally considered to be inhibitory, prior research has shown that facilitations are observed on inhibited neurons (e.g., [7]). The study focused on high spatial frequencies, where amblyopic deficits are most pronounced, and assessed contrast sensitivity before and after both single and repeated daily cTBS sessions. The results showed significant improvements in contrast sensitivity to high spatial frequencies in the amblyopic eye; cumulative effects were found to stabilize after two sessions, i.e., repeated administration of cTBS did not lead to larger effects but did result in more sustained improvements. The gains persisted for up to 78 days and were specific to the amblyopic eye, with no changes observed in the non-amblyopic eye. Moreover, no changes in global cortical excitability (assessed by phosphene threshold) were observed. These findings highlight the value of designing treatment protocols, which are driven by a theoretical model of the neural underpinnings of the condition; they also demonstrate the contribution towards understanding the mechanisms.

A recent study [10] investigated the effect of repetitive transcranial magnetic stimulation (rTMS) on visual function in 148 adult patients with anisometropic amblyopia. The combination of rTMS and intraocular lens implantation (ICL) significantly improved visual acuity and random dot stereopsis at three months compared to ICL alone. In this case, the intraocular lens acted as a modulator of the visual cortex’s long-term state, thereby influencing the effect of rTMS. Resting-state fMRI confirmed that the combined treatment modulated neuronal activity and enhanced visual cortical neuroplasticity, offering a non-invasive method for improving treatment efficacy. Further research is needed to determine the factors determining the stability and longevity of the rTMS effects, and to investigate potential methods for enhancing these effects. Besides, the existing literature on the effect of repetitive TMS in adults with amblyopia is rather sparse (see [45] for a systematic review). While most published studies indicate significant improvements, this body of evidence may be subject to publication bias, with negative or inconclusive results possibly unpublished. Consequently, large, randomized, double-blind designs would be important to establish the true efficacy of this intervention.

### 5.2. Post-Stroke Hemianopia

Hemianopia is a visual field loss affecting half of the visual field in both eyes, caused by damage to the brain’s visual pathways following a stroke. Over the past 40 years, there have been numerous attempts to develop rehabilitation protocols, with mixed success (e.g., [46,47]). Visual restoration training (VRT) involves reducing the size of the field defect by repeatedly presenting stimuli (over many sessions) in the patients’ blind field (either deep inside or on the border of the field defect) while the patient is asked to detect and discriminate them (e.g., [48]). While success has been variable in cases where psychophysically measured visual detection sensitivity in the blind field has improved (e.g., [49]), neuroimaging studies indicated the intact hemisphere in functional recovery. For example, in a study by [50], functional magnetic resonance imaging (fMRI) mapping revealed the development of representations of the recovered visual field in the intact visual cortex, more specifically in the visual motion-sensitive area V5/MT, in a region around the superior temporal sulcus and in retinotopic visual areas V1, V2, V3, and V3a. The effects are likely to result from the development of novel subcortical pathways to the intact hemisphere (cf. [51]).

Combining visual restoration therapies with non-invasive brain stimulation in a state-dependent manner has been shown to enhance their effectiveness. The logic is to use brain stimulation techniques to increase visual cortical excitability of the residual cortex, enhancing the neural responses evoked by stimuli presented in VRT. In effect, brain stimulation is used to bring the residual cortex, likely to be in a state of reduced excitability, into a state in which it is more likely to benefit from rehabilitation approaches (see [52] for a detailed discussion of this approach). In a single paired case study by Plow et al. [53], transcranial direct current stimulation (tDCS) was applied concurrently with VRT during one-hour sessions, three times per week, over three months. The patient receiving active tDCS with VRT showed significantly greater improvements in visual field expansion, fixation performance, vision-related daily activities, and quality of life compared to those receiving VRT with sham tDCS. However, no follow-up data for the sustainability of the effect is available.

Although brain stimulation research on cortical blindness using tDCS is promising, TMS also holds significant potential for facilitating visual recovery. As discussed earlier, TMS is particularly effective at enhancing weak neural responses, which are a hallmark of visual stimulation in the blind field. When applied at low intensity and timed closely with visual stimulation, TMS can amplify these weak responses (cf. [20]), strengthening neural activity and promoting plastic changes that support the formation of new neural representations. By repeatedly pairing visual stimuli in the blind field with precisely timed TMS pulses targeting regions where new representations are expected to emerge, this approach holds promise for accelerating visual recovery and improving adaptation in individuals with hemianopia.

A pilot study of Tiksnadi et al. [54] involved eight subjects with visual hemianopia due to ischemic stroke, with a median stroke onset of eight weeks. After ten consecutive rTMS sessions over two weeks, two cases showed very good improvement in the most affected side with VFI (visual field index) increases of 54% and 26%, while four cases had a mean improvement of 5%, and two cases experienced a regression of about 8.5%. On the lesser affected side, one patient improved by 43%, whereas the remaining cases showed an average increase of 5.57%, indicating high variability in the effects. The authors do not specify any follow-up data for these patients.

These examples demonstrate how consideration of the brain state associated with the clinical conditions is vital in designing an appropriate rehabilitation protocol. In addition to enhanced efficacy, they also contribute towards a mechanistic understanding of the condition itself. Similar to amblyopia, research on rTMS for hemianopsia is limited. Although published studies report some improvements, there is a potential for publication bias, as studies with negative or inconclusive results may remain unpublished. Therefore, conducting large-scale, randomized, double-blind trials would be the best approach to accurately determine the true effectiveness of this intervention in hemianopsia.

### 5.3. Recommendations for the Use of State-Dependency in the Clinical Domain

#### 5.3.1. Understanding and Assessing Brain State

Prior to initiating any brain stimulation protocol, an understanding of the patient’s individual brain state is necessary. This involves considering the specific neurological condition, its impact on cortical excitability, and any compensatory mechanisms that may be in play. Clinicians should utilize a combination of clinical assessments, patient history, and, where feasible, objective measures, such as resting-state EEG or cognitive tasks, to infer the current state of the targeted brain region. Recognizing that neurological disorders often alter brain states, compared to healthy individuals, clinicians should be cautious in directly translating stimulation parameters from studies conducted on healthy populations.

#### 5.3.2. Tailoring Stimulation Parameters

With a clearer understanding of the patient’s brain state, stimulation parameters (intensity, frequency, timing, and location) must be tailored to the individual. The principle of state-dependency implies that the same stimulation protocol can have different effects depending on the underlying brain state. For example, a region with reduced excitability may require higher stimulation intensities or frequencies to achieve the desired facilitatory effect. Conversely, in areas with heightened excitability, lower stimulation intensities may be sufficient or even necessary to avoid inducing paradoxical inhibitory effects. Monitoring patient response through behavioral assessments and, ideally, neurophysiological measures is critical to refine and optimize stimulation parameters throughout the course of treatment.

## 6. Discussion

State-dependent TMS offers a way to move beyond simply “disrupting” brain activity. By targeting specific neural populations based on their pre-existing activity, state-dependent TMS can selectively amplify or suppress the signals that carry information about prediction errors, thus influencing the refinement of internal models within the visual cortex. It is possible to discuss the application of state-dependent brain stimulation within the context of the brain’s internal models of the environment. This theoretical framework posits that the brain is constantly generating and refining internal models of the world based on incoming sensory information [55,56]. In a more mathematical formulation, this approach is often referred to as predictive coding and uses a Bayesian approach to upgrade internal models (predictions).

State-dependent plasticity-inducing TMS paradigms can be understood as a means of reshaping the brain’s internal models and optimizing predictive accuracy. By pairing stimulation with specific sensory experiences or cognitive tasks, it may be possible to selectively strengthen connections that support more accurate predictions and reduce reliance on error correction. For example, the finding that TMS selectively impairs the detection of incongruent targets in visual priming experiments from [10] can be interpreted within a predictive coding framework as disrupting the neural processes involved in resolving prediction errors.

Precision weighting refers to the brain’s estimate of the reliability of sensory information or internal predictions, which influences how strongly these signals are weighted in the process of updating internal models. The shifting facilitatory-inhibitory range described by [13] may reflect changes in the precision weighting assigned to sensory input and internal predictions. When excitability is reduced, the brain may increase the weight of incoming information (the gain) to compensate. This results in shifting the facilitatory range towards higher stimulation intensities.

Within a predictive coding context, the above-discussed ability of TMS to enhance contrast sensitivity in amblyopia suggests that the visual deficits are not due to an absence of functional circuits. Rather, the internal models of the visual system are somehow unbalanced, and brain stimulation offers a way to improve the model. As to the effectiveness of combined VRT and brain stimulation in treating hemianopia, it may be attributed to enhancing the precision weighting of sensory input in the damaged or recovering visual cortex, allowing the brain to more effectively utilize the available information and remap areas.

Thus, the predictive coding framework in its simple conceptual formulation provides a reasonable theoretical foundation for understanding the mechanisms underlying state-dependent brain stimulation and for guiding future research in this area. By focusing on the interplay between internal models, prediction errors, and precision weighting, it may be possible to develop more targeted and effective interventions for a wide range of neurological and psychiatric conditions.

## 7. Conclusions

State-dependent brain stimulation has emerged as a promising approach for enhancing the therapeutic potential of non-invasive brain stimulation techniques. This methodology tailors stimulation to the brain’s dynamic state, providing a pathway to more precise, effective, and enduring interventions across various neurological conditions. By conceptualizing stimulation effects as an interaction between external parameters and the brain’s ongoing activity, this framework allows for targeted modulation of neural circuits. Examples such as amblyopia and post-stroke hemianopia demonstrate how state-dependent paradigms can effectively facilitate plasticity in the visual cortex, offering new avenues for treatment and rehabilitation. Future research should focus on developing more sophisticated methods for assessing brain state, modifying it for a more targeted analysis, refining stimulation parameters to optimize individualized treatment protocols with more focus on the long-term clinical benefits of state-dependent interventions. Ultimately, this multi-directional approach to TMS will pave the way for more personalized neurorehabilitation, possibly enhancing the brain’s inherent plasticity to restore function and improve the quality of life for individuals with neurological and neurosensory disorders. The conceptual framework of internal models and predictive coding may provide a useful foundation for understanding the mechanisms underlying state-dependent brain stimulation and for guiding future research.
